# Weibo or WeChat? Assessing Preference for Social Networking Sites and Role of Personality Traits and Psychological Factors

**DOI:** 10.3389/fpsyg.2018.00545

**Published:** 2018-04-27

**Authors:** Juan Hou, Yamikani Ndasauka, Xuefei Pan, Shuangyi Chen, Fei Xu, Xiaochu Zhang

**Affiliations:** ^1^Department of Philosophy, Anhui University, Hefei, China; ^2^School of Humanities and Social Science, University of Science and Technology of China, Hefei, China; ^3^Department of Philosophy, Chancellor College, University of Malawi, Zomba, Malawi; ^4^Anhui Mental Health Center, Hefei, China; ^5^CAS Key Laboratory of Brain Function and Disease, School of Life Science, University of Science and Technology of China, Hefei, China

**Keywords:** social networking sites, Weibo, WeChat, preference, personality traits

## Abstract

Research trying to understand individual difference in the use of different social networking sites (SNSs) is minimal. In the present study, we collected data from 714 college students in China (273 males) to assess how personality traits and psychological factors relate to excessive use of WeChat and Weibo. We found that excessive use of Weibo and WeChat correlated positively with neuroticism, loneliness, and external locus of control and negatively with agreeableness, social support, and social interaction. Furthermore, people that scored high on loneliness, lack of social support, and poor social interaction skills excessively used Weibo more than WeChat. These results entail that by fulfilling different needs, WeChat and Weibo attract different kinds of people; significant lesson for future development of SNSs.

## Introduction

The internet is an essential component of our life, which influences many aspects of human behavior. Through the internet, people play games, shop, communicate, socialize, search-for, and spread information (Amichai-Hamburger and Ben-Artzi, 2000). Over the years, social networking sites (SNSs) have become the most popular and fastest web sites for information dissemination and private social interaction (Hughes et al., 2012). Popular SNSs such as Facebook and Twitter in US and WeChat and Weibo in China, allow individuals to express themselves, exchange information, and socialize. These sites attract and host millions of users.

An interesting question arises: considering that most SNSs attract millions of users, do these different sites attract different users? Joinson (2004) pointed out that internet behavior is a product of both the user and the specific tool, such that individual differences and personality can influence computer-media choices (Amiel and Sargent, 2004; Ryan and Xenos, 2011; Hou et al., 2014). Just as “Uses and Gratifications Theory” points out that “the audience is conceived as active” (Katz et al., 1974, p. 20). This means that, a user will use specific text to gain the valuable knowledge that they are seeking from a program or text (Katz et al., 1974). Because in mass communication much initiative to link media choice lies with users (Katz et al., 1974). This implies that people use the media to fulfill individual's specific different needs and gratifications.

Therefore, under the “Uses and Gratification Theory,” which seeks to understand the use of media from the perspective of the individual and not the media, individual experiences are important factors in determining the choice of SNSs. So, this study seeks to understand if particular individual differences are related to the preference for the excessive use of SNSs. We investigated the role of individual difference in the excessive use of SNSs in this study. This is an exploratory study that aimed to find out the relationship between personality traits/psychological factors and excessive use of Weibo and WeChat. Thus, we assessed how Big-Five personality traits, loneliness, social support, external locus of control, and social interaction relate to the excessive use of two largest SNSs in China- WeChat and Weibo.

### WeChat and Weibo

WeChat is one of the most popular SNSs in China and currently boosts of over 600 million active users worldwide (CNNIC, 2015). WeChat is a cross-platform communication application combining popular features of Facebook and WhatsApp (Wu, 2014). It allows users to create a profile, search for friends or find new friends within one's geographical location. On this profile, users can send instant text messages, voice notes and make free voice calls. Further, WeChat allows users to post information, pictures and videos of interest, and comment on friends' posts. All of these features make WeChat popular for online socializing.

Another popular SNS in China is Weibo, created in 2009, and has more than 204 million users (CNNIC, 2015). Weibo allows users to post 140-character information. Similar to Twitter, but unlike WeChat, Weibo focuses on sharing of opinions and information exchange rather than on social interaction (Kwak et al., 2010); and offers some anonymity in online communication (Huberman et al., 2008). Weibo does not need users to post their private information to find “friends” and it focuses less on “who you are” and more on “what you say” (Huberman et al., 2008).

The reduction of social pressure brought about by anonymity (Hughes et al., 2012) may mean different motivation for using Weibo from WeChat. Further, a previous study of interest found that only 2 out of 10 interviewed people had used WeChat to search for new friends (Hou et al., 2017). This entails that most people use WeChat to keep in touch with friends they made in real life. It is hence expected that these differences will be evident in the relationships between individual traits and excessive use of WeChat and Weibo.

### Personality

A number of personality traits appear to be associated with the use of SNSs. The Big-Five personality test, developed by Goldberg (1999), is the most commonly used model in investigating the relationship between internet use and individual personality (Landers and Lounsbury, 2006; Ehrenberg et al., 2008). The Big-Five personality test consists of five factors: neuroticism, extraversion, openness, agreeableness, and conscientiousness (McCrae and John, 1992). Several of the factors are associated with problematic use of online social media, such as blogs (Guadagno et al., 2008) and SNSs (Ross et al., 2009; Amichai-Hamburger and Vinitzky, 2010; Correa et al., 2010).

Neuroticism is characterized by anxiety, hostility, depression, self-consciousness, impulsivity, and fragility. Individuals who are low in this trait tend to be more stable and emotionally resilient. Butt and Phillips (2008) found that people with high scores in neuroticism use the Internet frequently, spent more time on Facebook (Ryan and Xenos, 2011) and instant messenger (Correa et al., 2010). Neurotics prefer to use the Internet to relieve loneliness and find a sense of group belonging (Amichai-Hamburger and Ben-Artzi, 2003; Butt and Phillips, 2008). In the current study, we hypothesized that those with high scores in neuroticism will excessively use Weibo more than WeChat (H1).

Extraversion is characterized by excitability, sociability, talkativeness, assertiveness, and high amounts of emotional expressiveness. People who are high in extraversion (also called extroverts) are like to be in touch with people, are full of energy and often feel positive emotions. People who are low in extraversion (also called introverts) are quiet, cautious, and don't like excessive contact with the outside world. Extraverts have been found to be excessive users of instant messaging and SNSs (Correa et al., 2010). They have more friends online (Amichai-Hamburger and Vinitzky, 2010) and tend to make even more friends offline (Ross et al., 2009). Thus, extraverts like socializing, but they don't take online socializing as a substitute for real life social interaction. Thus, extraverts use SNSs mainly for social enhancement. People with only a few offline contacts compensate for their introversion, low self-esteem, and low life-satisfaction by using Facebook for online popularity (Ellison et al., 2007; Barker, 2009; Pollet et al., 2011). In this study, we hypothesized that extraversion will positively correlate more with excessive use of WeChat than Weibo (H2).

Openness has the characteristics of imagination, aesthetics, rich emotions, differences, creativity, intelligence, etc. People who score high in this trait prefer abstract thinking and have a wide range of interests. People who score low in this trait are practical, preferring conventions, and more traditional and conservative. Individuals who score high on openness have been found to excessively use instant messaging and SNSs (Correa et al., 2010). They have wide interests and curiosity (McCrae and Costa, 1987), so they prefer to use Internet for information seeking (McElroy et al., 2007). So, since Weibo can provide more new information than WeChat, in this study, we expected that the excessive use of Weibo would be more in open people than WeChat (H3).

Agreeableness has qualities such as trust, altruism, outspokenness, compliance, modesty, empathy, and so on. People who score high in agreeableness are considerate, friendly, generous, and helpful and willing to give up their own interests for others. Agreeableness has been found to be unrelated to Internet and SNSs' use in many studies (Amichai-Hamburger and Vinitzky, 2010; Correa et al., 2010). However, Ross et al. (2009) pointed out that less agreeable people interact more online and take Internet as a tool to improve social skills and build friendships. La et al. (2009) found that females scoring high on this trait posted significantly more pictures than females scoring low, with the opposite being true for males. In the current study, we hypothesized that agreeableness will negatively correlate more with excessive use of Weibo than WeChat (H4).

Conscientiousness shows the characteristics of competence, impartiality, coherence, due diligence, achievement, self-discipline, discretion, and restraint. Those who score high on conscientiousness are responsibility, dedication to work, and seriousness. Numerous studies have found significant negative correlation between conscientiousness and SNSs' use time (Amichai-Hamburger and Vinitzky, 2010; Ryan and Xenos, 2011). Butt and Phillips (2008) suggested that conscientious people do not allow SNSs to disrupt their important work. They may prefer Twitter to Facebook because “tweets” are limited to 140 characters, which mean just a temporal distraction for them (Hughes et al., 2012). In addition to this, people with high conscientiousness were found to have significantly more friends and to upload significantly fewer pictures than those scoring low on this personality trait (La et al., 2009). Thus, conscientious people tend to cultivate their online and offline contacts more without the necessity to share too much personal information publicly. We hence hypothesized that conscientiousness will negatively correlate with the excessive use of WeChat (H5), but will not correlate with the excessive use of Weibo.

Ross et al. (2009) argued that the Big Five might be too broad when assessing individual differences in SNS usage. Taking into account the characteristics of SNSs, this study included different psychological factors, namely loneliness, social support, external locus of control, and social interaction.

### Loneliness and social support

Loneliness is considered as one of the most important predictors of internet addiction (Baumeister et al., 2005; Wang, 2006; Bozoglan et al., 2013). Lonely people usually report less support from their social network in real life (Routasalo et al., 2006). McKenna et al. (2002) found that people who feel lonely are more likely to prefer online social interactions than face-to-face settings (Clerkin et al., 2013; Ye and Lin, 2015). Further, people with low real life social support and high virtual social support tend to draw support from online communication (Yeh et al., 2008).

Admittedly, causal direction of this relation is not clear. There is a two-way relationship between loneliness/social support and SNS use. On the one hand, lonely individuals are attracted to SNS to relieve loneliness or get support; on the other hand, excessive use of SNS has been found to increase sense of loneliness (van den Eijnden et al., 2008). Further, through internet communication, particularly communication with known people, lonely people can increase sense of social support (Shaw and Gant, 2002). Based on socializing features of WeChat, which mainly features people one interacts with in real life and the anonymity of Weibo, we hypothesized that lonely people and those with lack of real life social support will excessively use Weibo more than WeChat (H6, H7).

### External locus of control and social interaction

Locus of control refers to the extent to which individuals believe that they can control things that affect them (Rotter, 1966). People with high external locus of control believe that their lives are controlled by luck, fate and chance (Ndasauka et al., 2016) and not by their effort or ability. Such people are more likely to engage in problematic internet use (Chak and Leung, 2004), have more online social interaction (Koo, 2009; Ye and Lin, 2015) and have low social interaction skills (Cloitre et al., 1992; Stipek, 1993). With diminished social interaction skills, they prefer communicating through the internet, where they can contact with others without face-to-face interaction (Ndasauka et al., 2016). In the present study, we expected that external locus of control will positively correlate with excessive use of both WeChat and Weibo (H8). We also hypothesized that social interaction in real life will negatively correlate with excessive use of Weibo more than WeChat (H9).

## Methods and materials

### Participants

Total number of participants was 714; 273 males (38.2%), 441 females (61.8%), and were recruited from 3 college campuses in Anhui province, East China. The mean age was 19.8 years (*SD* = 1.3) ranging from 17 to 21, and 11 participants were under 18 years old.

### Ethics statement

The study was approved by the Human Research Ethics Committee of the University of Science and Technology of China (USTC). All participants gave consent to participate in the study and principles expressed in the Declaration of Helsinki were closely followed. Participants were undergraduate students.

We did not obtain informed consent from the next of kin, caretakers, or guardians on behalf of the minors/children (under 17 years) enrolled in our study. These young college students were considered to have comparable intelligence and ability to adult students, and able to take charge of their behaviors. According to the General principles of the Civil Law of the People's Republic of China; “A minor aged 10 or over shall be a person with limited capacity for civil conduct and may engage in civil activities appropriate to his age and intellect; in other civil activities, he shall be represented by his agent ad litem or participate with the consent of his agent ad litem” (Article 12, Chapter II). Therefore, we obtained the same consent from these participants between 17 and 18 as those above 18 years old, which was also approved by the Human Research Ethics Committee of the University of Science and Technology of China (USTC). Small gifts (keychain and nail-cutter of not more than $1.5) were given as incentive to participate in the study. Written informed consent was obtained from all participants.

### Measures

#### Demographic data

Participants answered two questions regarding their gender and age. We also asked participants to rate themselves with regard to their preference of WeChat and Weibo. They were asked to choose one between Weibo and WeChat by stating which one they preferred.

#### WeChat excessive use scale (WEUS)

The scale was developed to assess excessive use of WeChat (Hou et al., 2017). It includes items such as “I check my WeChat before something else that I need to do,” “I have used WeChat to relieve of loneliness and stress,” and “There are times when I would rather play on WeChat than go out with my friends.” The 10-item scale showed good internal consistency in the initial study, with Cronbach's alpha of 0.907. In the current study, the Cronbach's alpha was 0.899. The scale is scored on a Likert-type scale ranging from 1 (*never*) to 5 (*always*).

#### Microblog excessive use scale (MEUS)

The scale was developed to measure excessive use of Weibo (Hou et al., 2014). It includes items such as “How often do you find yourself saying “just a few more minutes” when using microblogs?,” “How often would you try to increase your followers unconsciously by all means?,” and “How often do you feel depressed, moody, or nervous when you are off microblogs?” MEUS has 10 items rated on a 6 point Likert scale from “1 = *never*” to “6 = *always*.” In the current study the scale showed good internal consistency with Cronbach's alpha of 0.908.

#### Big five personality questionnaire

We used a 60-item personality questionnaire developed by Leung (2011) to assess five different personalities, namely, Neuroticism, Extraversion, Openness, Agreeableness, and Conscientiousness. Each factor featured 12 items rated on 5-point scale ranging from *strongly disagree* to *strongly agree* (McCrae and Costa, 1991). Four personalities factors of Neuroticism (α = 0.708), Extraversion (α = 0.648), Agreeableness (α = 0.573), and Conscientiousness (α = 0.697) showed adequate to good reliability and internal consistency in our sample. However, Openness factor showed poor reliability and internal consistency (α = 0.371). As such, this personality factor was not included in our analyses.

#### UCLA loneliness scale

This scale, originally developed in 1978 by Russell, Peplau, and Ferguson, has 20 items rated on a 4-point scale from “1 = *never*” to “4 = *often*.” In the current study, the Cronbach's alpha was 0.760.

#### Social support scale

We used the Interpersonal Support/Social Support Scale; a 12-item measure of perceptions of social support. This measure is a short version of the original ISEL (40 items; Cohen and Hoberman, 1983). Items are rated on a 4-point scale ranging from “*definitely true*” to “*definitely false*.” In the current study, the Cronbach's alpha was 0.770.

#### Locus of control scale (LOC)

We used the multidimensional locus of control scale developed by Levenson (1981). The scale has three dimensions: Internal scale, which measures internal locus of control; Powerful Others scale and Chance scale, which measure external locus of control. In this study, we utilized the latter two scales to measure external locus of control. The scale had 16 items and were scored on a 6-point Likert scale from “1 = *strongly disagree*” to “6 = *strongly agree*.” The scale showed adequate internal consistency in our study (α = 0.738).

#### Social interaction scale (SIS)

We employed the Social Interaction Scale developed by Yan (2011). The Social Interaction Scale contains 24 questions divided into two parts namely- Real Life Scale (14 items) and Online Scale (10 items). In our study, we only used the Real Life Scale to measure interpersonal communication with classmates, friends, parents, and other people in real life. The items are rated on a 4-point Likert scale from “1 = *never*” to “4 = *always*.” In our study, the Real Life scale showed good internal consistency and reliability (α = 0.808).

### Data analysis

We analyzed the data using the statistics software package SPSS 23.0. We calculated correlations between the scales using Pearson's *r* (Pearson product-moment correlation coefficient). To test significant differences of different levels in total scores, we used the Kruskal–Wallis test (Kruskal–Wallis one-way analysis of variance) and *t*-tests. We also used Scheffe's *post-hoc* tests to analyze significant differences between different levels. Further, we used a method by Lee and Preacher (2013) to calculate the difference in correlations between WEUS ↔ personality traits and MEUS ↔ personality traits. The significance level in this study was *p* ≤ 0.05.

## Results

### Weibo vs. WeChat

With regard to preference, 386 participants (females = 261) reported that they preferred Weibo to WeChat, while 328 participants (females = 180) reported that they preferred WeChat to Weibo. We used the chi-square test to calculate the gender proportion, and there was significant difference between the usage proportion of Weibo and WeChat (χ^2^ = 12.184, *p* < 0.001), with more females preferring Weibo to WeChat.

We analyzed the correlation between MEUS and WEUS. Results showed significant positive correlation between the two variables (*r* = 0.462, *p* < 0.001).

### Weibo, WeChat, conscientiousness, extraversion, neuroticism, and agreeableness

We analyzed the correlation between MEUS and the four personality traits (see Table 1). MEUS positively correlated with Neuroticism (*r* = 0.173, *p* < 0.001), negatively with Agreeableness (*r* = −0.234, *p* < 0.001), and Conscientiousness (*r* = −0.083, *p* = 0.026), but did not significantly correlate with Extraversion (*r* = 0.007, *p* = 0.852).

**Table 1:** Correlation between MEUS/WEUS and personality traits, loneliness/social support, and social interaction skills in real life/ external locus of control.

	**Neuroticism**	**Extraversion**	**Agreeableness**	**Conscientiousness**	**Loneliness**	**Social support**	**Social interaction skills in real life**	**External locus of control**
MEUS	0.173^***^	0.007	−0.234^***^	−0.083^*^	0.139^***^	−0.111^**^	−0.084^*^	0.179^***^
WEUS	0.118^**^	0.057	−0.153^***^	−0.025	0.025	−0.048	−0.008	0.208^***^

* *p < 0.05*,

** *p < 0.01*,

*** *p < 0.001*.

**Figure 1:**
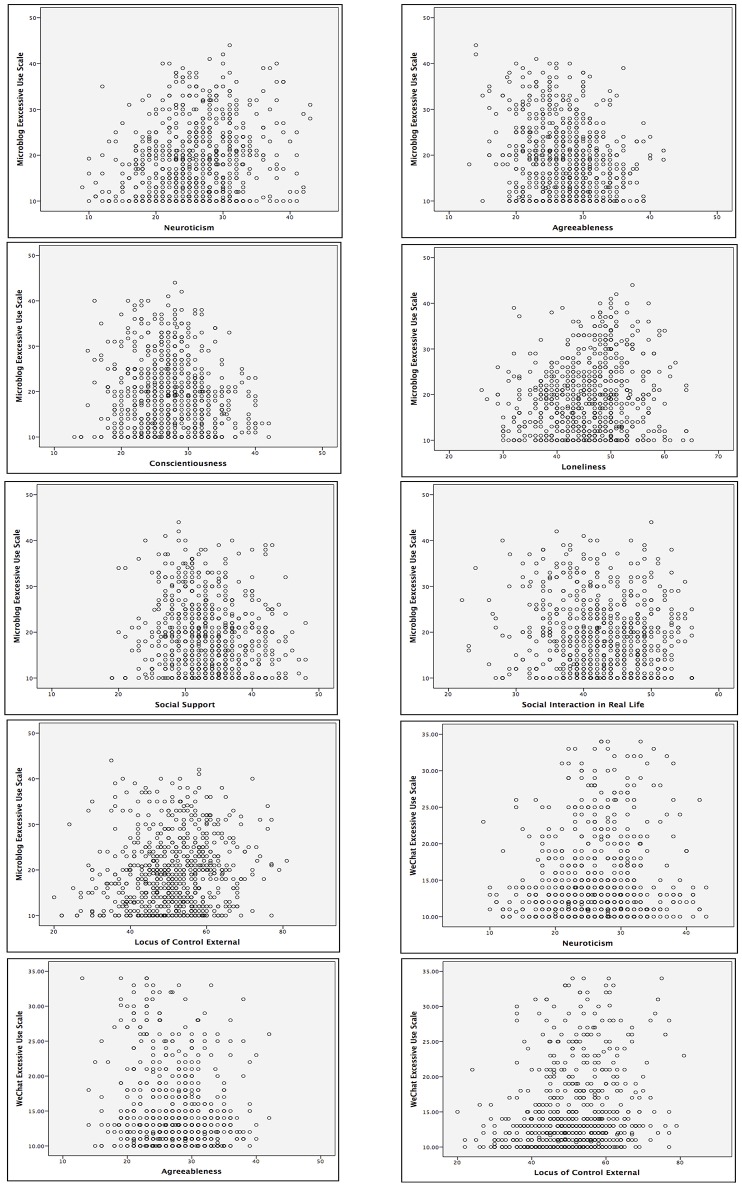
Scatter plot of the correlation between MEUS/WEUS and personality traits, loneliness/social support and social interaction skills in real life/ external locus of control.

We then analyzed the correlation between WEUS and the four personality traits (see Table 1). WEUS positively correlated with Neuroticism (*r* = 0.118, *p* < 0.001), negatively with Agreeableness (*r* = −0.153, *p* < 0.001) but did not significantly correlate with Conscientiousness (*r* = −0.025, *p* = 0.503) and Extraversion (*r* = 0.057, *p* = 0.128). For the significant correlations, we also draw the scatter plot on the relationship between MEUS/WEUS and the four personality traits (see Figure 1).

Further, we found significant difference between correlations of MEUS ↔ Agreeableness and WEUS ↔ Agreeableness (*z* = −2.14, *p* = 0.033). These results were consistent with H4 and H1, H2, H3, H5 were not supported.

### Weibo, WeChat, loneliness, and social support

Our results showed that loneliness positively correlated with MEUS (*r* = 0.139, *p* < 0.001) but did not significantly correlate with WEUS (*r* = 0.025, *p* = 0.508; see Table 1). We analyzed the difference between these two correlations and found that correlation between loneliness and MEUS was significantly higher than correlation between loneliness and WEUS (*z* = 2.931, *p* = 0.003).

Further, we found that social support negatively correlated with MEUS (*r* = −0.111, *p* = 0.003), but did not significantly correlate with WEUS (*r* = −0.048, *p* = 0.200; see Table 1). For the significant correlations, we also draw the scatter plot on the relationship between MEUS/WEUS and loneliness/social support (see Figure 1).

We then analyzed the difference between the two correlations and found that the two correlations were not significantly different (*z* = 1.627, *p* = 0.104). These results were consistent with H6 and H7 is not supported.

### Weibo, WeChat, external locus of control, and social interaction in real life

We analyzed the correlation between MEUS and external locus of control/real life social interaction (see Table 1). MEUS correlated positively with external locus of control (*r* = 0.178, *p* < 0.001), and negatively with real life social interaction (*r* = −0.084, *p* = 0.024).

We then analyzed the correlation between WEUS and the two variables (external locus of control and real life social interaction; see Table 1). WEUS correlated positively with external locus of control (*r* = 0.208, *p* < 0.001), but did not significantly correlate with real life social interaction (*r* = −0.008, *p* = 0.822). For the significant correlations, we also draw the scatter plot on the relationship between MEUS/WEUS and external locus of control/social interaction in real life (see Figure 1).

While the correlation of MEUS and external locus of control did not significantly differ with correlation of WEUS and external locus of control (*z* = 0.79, *p* = 0.429), correlation of MEUS and real life social interaction was significantly higher than correlation of WEUS and real life social interaction (*z* = 2.243, *p* = 0.025). These results were consistent with H9 and H8 is not supported.

### Correcting for multiple comparisons

Applying the Bonferroni correction, we divided *p* = 0.05 by the number of tests (10) to get the Bonferroni critical value. This resulted in change of *p*-value to *p* < 0.005 to be significant. Under that criterion, all tests were found to be significant except for correlation between MEUS and Conscientiousness whose *p* = 0.026 and MEUS and real life social interaction whose *p* = 0.024.

## Discussion

The aim of the current study was to investigate some of the individual differences associated with the use of the two largest SNSs in China- Weibo and WeChat. We found that different personality traits were influential in explaining the excessive use of the two SNSs, and some correlations between these traits and Weibo and WeChat were also significantly different.

### Weibo vs. WeChat

From the item- “which one do you prefer, Weibo or WeChat?” results showed that more females preferred Weibo to WeChat than males. This is consistent with previous studies that females scored significantly higher on the Weibo scale than males (Hou et al., 2014), and women are more likely to use Twitter than men (Smith and Rainie, 2010). By broadcasting to everyone, Weibo allows young female students to express themselves to and seek attention from a larger audience than they would on WeChat, which is limited in its broadcasting and sharing of moments.

Also, we found that Weibo and WeChat had significant moderate correlation with each other. These results may mean that users of one also tend to use another. However, the results may also entail that there is some difference between Weibo and WeChat.

### The use of Weibo and WeChat

#### Personality differences in the use of Weibo and WeChat

Neuroticism positively correlated with excessive use of both Weibo and WeChat. This result is consistence with previous studies that neurotic people are more likely to use SNSs for socializing (Amichai-Hamburger and Ben-Artzi, 2000; Butt and Phillips, 2008). However, contrary to our hypothesis, there was no significant difference between correlations of neuroticism ↔ use of Weibo and neuroticism ↔ use of WeChat. These results imply that neurotic people use both two SNSs in similar manner. One factor that may help explain this is Weibo's anonymity and other anonymity features of WeChat, like “Shake” and “drift bottle.” This allows neurotics to interact with people online, because these forms of interaction do not require face-to-face contact. As Amichai-Hamburger et al. (2002, p. 127–128) reported: “It would appear that the social services provided on the Internet, with their anonymity, lack of need to reveal physical appearance, rigid control of information revealed in the interaction… provide an excellent answer to people who experience great difficulty in forming social contacts due to their introverted personality.”

Agreeableness negatively correlated with both Weibo and WeChat. This result also is supported by Ross et al. (2009) that less agreeable people are inclined to use SNSs more often and sometimes in an excessive way. Further, consistent with our hypothesis, we found significant difference between correlations of agreeableness ↔ use of Weibo and agreeableness ↔ use of WeChat. Thus, less agreeable people are more likely to excessively use Weibo than WeChat. Since less agreeable people are considered less friendly, they may find Weibo a better alternative to fulfill their social needs than WeChat. Although they use WeChat, Weibo provides them a wider scope of social fulfillment because they can interact with many people with whom they are not friends in real life.

Conscientiousness negatively correlated with the use of Weibo in our study, but did not significantly correlate with WeChat. These results are a direct contrast to our hypotheses. This may be due to the other attributes of both Weibo and WeChat. Weibo is a half-open platform, and for highly conscientious people, it may increase sense of insecurity, which may not be the case with WeChat because they may feel in control of their friends' circle and hence feel secure to post pictures and socialize without fear. A previous study of interest found that despite having more friends than those scoring low in the trait, conscientious people tend to upload significantly fewer pictures on Weibo (Amichai-Hamburger and Vinitzky, 2010).

#### Loneliness and social support differences in the use of Weibo and WeChat

Loneliness positively correlated with the use of Weibo while social support negatively correlated with the use of Weibo. Surprisingly however, the two factors did not significantly correlate with WeChat. Furthermore, we found significant difference between correlations of the two psychological factors ↔ use of Weibo and the psychological factors ↔ use of WeChat. The results suggest that individuals who are lonely and people who lack social support in real life tend to use Weibo more than WeChat. Thus, because Weibo is half-open platform, people can engage in identity experimentation, which brings more gratification to lonely people than those or not lonely (Leung, 2011). Further, the open and anonymity attributes of Weibo (Hughes et al., 2012) offer people an opportunity to jump out of the real-life circle of friends. Through Weibo, lonely people can make new friends, seek novelty and information of interest. This is unlike WeChat; whose posts are mainly from relatively the same people one interacts with in real life. As such, WeChat may bring the same isolated feeling for people that lack social support and feel lonely.

#### External locus of control and social interaction in real life differences in the use of Weibo and WeChat

Our results showed that excessive use of both Weibo and WeChat is associated with external locus of control. Thus, people who have faith in that environment causes their life events, excessively use Weibo and WeChat. These findings are similar to results of previous studies (Karatas and Tagay, 2012; Ndasauka et al., 2016). Thus, externals are lonelier (Hojat, 1982) and feel less confident in control of their lives and behaviors (Ye and Lin, 2015) hence they excessively use WeChat and Weibo.

Further, we also found negative correlation between Weibo and social interaction in real life. However, we found no significant difference between correlations of problematic use of Weibo ↔ social interaction in real life and the use of WeChat ↔ social interaction in real life. This entails that people who spend less time in socializing in real life or lack social skills in real life tend to choose online social interaction on Weibo. Compared to WeChat, Weibo provides them a bigger “circle of friends,” where they can practice social skills with strangers. In addition, the reduction of social pressure on Weibo (Hughes et al., 2012) may be the one of the factors that attracts people with poor real life social skills.

## Conclusion and limitations

Overall, the study investigated how individual difference are associated with the use of Weibo and WeChat. Results demonstrate that personality traits are linked to excessive use of Weibo and WeChat. We found that personality such as neuroticism, loneliness, and external locus of control had positive correlations with excessive use of Weibo and WeChat, while agreeableness, social support, and social interaction negatively correlated to excessive use of Weibo and WeChat. Furthermore, we compared the difference between correlations of the personal traits and excessive use of Weibo and WeChat, and we found that lonely people, people that lack social support and those with poor social interaction skills tend to excessively use Weibo more than WeChat. These results are pertinent because they entail that people who experience loneliness or social frustration in real life choose sites that impose less social pressure to relieve loneliness and maybe gain confidence for real life social interaction.

The study had some limitations that merit consideration. Firstly, the selected respondents were only from Eastern China, and the sample representation may not be completely correct. Secondly, the study exclusively used self-report questionnaires in data collection. Due to social desirability or understanding problem, self-reporting sometimes affects reliability and validity of the answers. Therefore, in future research, we need to combine a variety of research methods to draw a complete picture of the use of Weibo and WeChat in China.

## Relevance and contribution of study

Although WeChat is considered as a more socializing platform than Weibo, which is viewed as platform for sharing information, our study has shown that people scoring high on loneliness and neuroticism are more likely to use Weibo than WeChat. The results of this study add to the uses and gratification theory, which states that people engage in some activity, in this case social networking, to meet certain psychological needs. As such, by meeting different needs of people, WeChat and Weibo tend to attract different kinds of people.

People using WeChat tend to mainly transfer their offline social interaction with friends to online environment. For people lacking social support and social interaction skills, transferring to WeChat does not often meet their social needs in real life. So, when dealing with people experiencing social problems, it is more meaningful to provide them with a broader social space and open platform than to let them practice their social skills with known friends. As such in helping people who are struggling with excessive use of Weibo, WeChat, or other social network applications, it is important to focus on improving their social skills, and reducing their social pressure and loneliness.

Further, medical practitioners dealing with people struggling with excessive or addictive use of SNSs should pay attention to the particular sites in which their patients are overusing. This is because, as shown in this study, different people are attracted and motivated to use different SNSs in that those with some psychological traits are more likely to use one more than another or others. Finally, these results should encourage SNSs developers to rethink the function of their sites. When developing social networking platforms, developers should consider how best to meet psychological needs of different groups.

## Author contributions

JH, YN, XZ, and FX: Conceived and designed the experiments; JH, YN, XP, and SC: Performed the experiments; JH, YN, XP, and SC: Analyzed the data; JH, YN, XP, and SC: Contributed reagents, materials, analysis tools; JH, YN, and XZ: Wrote the paper; JH, YN, XZ, and FX: Discussed the result; JH, YN, XZ, and FX: Final approval of the version to be published.

### Conflict of interest statement

The authors declare that the research was conducted in the absence of any commercial or financial relationships that could be construed as a potential conflict of interest.
